# The cultural evolution and ecology of institutions

**DOI:** 10.1098/rstb.2020.0047

**Published:** 2021-07-05

**Authors:** Thomas E. Currie, Marco Campenni, Adam Flitton, Tim Njagi, Enoch Ontiri, Cedric Perret, Lindsay Walker

**Affiliations:** ^1^ Human Behaviour and Cultural Evolution Group, Centre for Ecology and Conservation, University of Exeter, Penryn Campus, TR10 9FE, UK; ^2^ Tegemeo Institute of Agricultural Policy and Development, Egerton University, Nairobi, Kenya; ^3^ Simply Green Worldwide, Nairobi, Kenya

**Keywords:** cultural evolution, institutions, political complexity, driven trend, barrier effects

## Abstract

Human societies are structured by what we refer to as ‘institutions’, which are socially created and culturally inherited proscriptions on behaviour that define roles and set expectations about social interactions. The study of institutions in several social science fields has provided many important insights that have not been fully appreciated in the evolutionary human sciences. However, such research has often lacked a shared understanding of general processes of change that shape institutional diversity across space and time. We argue that evolutionary theory can provide a useful framework for synthesizing information from different disciplines to address issues such as how and why institutions change over time, how institutional rules co-evolve with other culturally inherited traits, and the role that ecological factors might play in shaping institutional diversity. We argue that we can gain important insights by applying cultural evolutionary thinking to the study of institutions, but that we also need to expand and adapt our approaches to better handle the ways that institutions work, and how they might change over time. In this paper, we illustrate our approach by describing macro-scale empirical comparative analyses that demonstrate how evolutionary theory can be used to generate and test hypotheses about the processes that have shaped some of the major patterns we see in institutional diversity over time and across the world today. We then go on to discuss how we might usefully develop micro-scale models of institutional change by adapting concepts from game theory and agent-based modelling. We end by considering current challenges and areas for future research, and the potential implications for other areas of study and real-world applications.

This article is part of the theme issue ‘Foundations of cultural evolution’.

## 1. Introduction

In comparison to other species, human societies are characterized by the fact that they create rules about what individuals are supposed to do in different situations. These rules and the social processes that generate and shape them make up what are known as ‘institutions’, which affect different aspects of our lives and fundamentally regulate and structure social interactions [1–5]. It is argued that this ability to create institutions helps bond societies together and underpins the fact that humans are able to cooperate, work together and perform collective behaviours with genetically unrelated individuals on a scale not seen in other species [2,6]. As well as acknowledging our species' capacity for cooperative behaviour, it is also important to recognize that across time and space there has been a great deal of variation in the scale at which human societies have been able to act collectively [7,8]. Here we argue that the ability of humans to construct systems involving culturally inherited rules of behaviour (institutions) helps shape cooperation and collective behaviour in humans, but that this hasn't been fully integrated into current evolutionary approaches to human behaviour. We argue that an evolutionary framework that integrates insights and approaches from the evolutionary human sciences (e.g. cultural evolutionary theory, behavioural ecology), and various more traditional social science and humanities disciplines (e.g. economics, political science, anthropology and archaeology) can be valuable in addressing questions about how institutions function and how they evolve. This will also involve not just applying existing theories and models from cultural evolution but will require us to expand and adapt our approaches to better handle the ways that institutions work, and how they might change over time. Here we present findings from previous and current research on these topics, and outline ways in which future research might progress.

### (a) What are institutions?

‘Institutions’ is a term (like many terms in the social sciences) that can have quite distinct meanings in different contexts and disciplines. Here we use the commonly held understanding that institutions are human-generated regulators of social interaction and refer to a wide variety of spheres of human society such as marriage, use of money, legal systems, systems of government, and ownership of property. Abstractly, we can think of institutions as involving proscriptions about what actions are permitted or must (or must not) be undertaken by certain defined individuals, under what conditions, and often the potential consequences for not following those rules [9]. For example, marriage is an institution that establishes a specific kind of lasting, inter-personal union between people. Although thought to be a cultural universal, different societies have different rules about the characteristics of people that are able to enter into marriage with each other (e.g. how old the participants must be, which groups they must come from, whether someone can be married more than once), and what rights or obligations being married has for those in the marriage and other relevant parties such as offspring and other family members. We also note that the term ‘institution’ is sometimes used to refer to specific groups, bodies or units (e.g. specific universities or banks), as these refer to particular groups and collections of people here we would refer to such entities as ‘organizations’.

All human societies have some form of institutions, but within and between cultures institutions can vary in the extent to which rules are implicit or explicit, written or unwritten, enforced or otherwise adhered to, and connected to or supported by other institutions. Even accepting this broad definition of institutions we can identify different schools of thought about how to conceptualize institutions. One view equates institutions with rules, such as the ones described by nations' laws and constitutions [5], while the other refers to institutions as stable, regular patterns of behaviour, or equilibria, e.g. everybody drives on the left side of the road because they expect others to do so and they prefer to avoid accidents [10] (for more detailed discussions of these distinctions, see [1,9,11]). In reality, both the ‘institutions-as-rules’ and ‘institutions-as-equilibria’ perspectives relate to important concepts which affect the knowledge that individuals have about different situations, the behaviour they exhibit in those situations, and how specific rules actually play out in practice. We return to this point in §1c in thinking about how these different aspects of institutions connect to cultural evolutionary theory. Unless specified, we do not make a distinction between these two forms when we use the term institution. Maintaining a rather broad and inclusive definition that is not tied to one specific theoretical or methodological approach allows us and other researchers to see connections across different disciplines and think about how these might be integrated in a synthetic evolutionary framework.

### (b) The function of institutions

Social living involves interacting with other individuals. While such interactions can provide benefits they often involve costs and can present challenges to individuals acting collectively. This can range from situations whereby individuals would benefit from coordinating their actions (e.g. driving on one agreed side of the road), to challenging social dilemmas where cooperating can provide group-level benefits but there are incentives to free-ride on the contributions or actions of others (e.g. agreeing fishing limits can facilitate the sustainable management of a commonly owned fishery, but this can be undone by individuals seeking to benefit in the short-term by taking more than an agreed share, such that the fishery collapses) [12]. At a functional level, institutional rules help facilitate potential solutions to persistent or recurring cooperation or coordination problems by helping to structure interactions to more frequently occur between certain individuals or by changing the pay-offs to different behaviours [5]. For example, institutions developed during medieval times provided a framework for enabling individuals to trade goods even at long distance. These institutions secured property rights required for sending goods abroad, and enforced contracts between individuals that would never meet [13]. By permitting, promoting or enforcing certain social interactions (but potentially not others) institutional rules can facilitate positive assortment, e.g. bring together those with a shared understanding in a coordination situation, or enabling cooperators to interact more easily with other cooperators in a collective action situation. This can happen in many ways such as creating conditions that enable monitoring of free-riding [14] or through facilitating reputational or signalling mechanisms [15,16]. Furthermore, by effectively changing the likely costs and benefits of different behaviours institutions can change the default ‘rules of the game’ from one in which pay-offs favour defection to one in which pay-offs favour cooperation [2]. To give a concrete ethnographic example, in irrigation systems in Nepal, communities have developed rules about collective maintenance of the irrigation system, and how much water can be taken in order to minimize free-riding [12]. If an individual is deemed to have not followed the rules then they receive a punishment: the transgressor has one of their cows placed in a pen in the middle of the village. Because the community is small, everyone in the village knows whose cow this is, and also other villagers are able to take milk from this cow. The cow is only released once a fine is paid. Therefore, someone who breaks the irrigation rules suffers directly by having to pay the fine and from losing the ability to milk the cow while it is in the pen. They also suffer reputational damage, which, in turn, may entail opportunity costs by inhibiting future interactions with other community members.

It should be clarified that not all institutions enforce cooperative or group-beneficial behaviours. First, institutions can exist without a clear function, in the same way that other cultural traits can be neutral or by-products of other adaptations [1,17]. Second, institutions can enforce socially detrimental behaviours if these institutions are badly designed [18], or designed by a minority for a minority [4]. For instance, institutions in Latin America initially developed by Spanish and Portuguese conquerors established a highly centralized, bureaucratic administration with rules that favoured the colonists, and led to wealth being extracted for their home populations in Europe. These institutions ended up promoting the role of personal relationships for business successes, arbitrary property rights and corruption, which hindered economic performance for Latin American countries in the longer run [5].

As institutions can strongly differ in their effects on different sections of a society, or in their ability to achieve certain goals, social science researchers working on institutions have examined the kinds of features that effective institutions seem to possess. For example, the work of Ostrom [19] has been key in understanding the functional design features of institutions that enable societies to successfully manage common-pool resources. We can draw analogies between these design features and more general evolutionary mechanisms (see also [20] for a similar approach but framed in terms of multilevel selection). For example, some principles relate to the pay-offs of cooperative behaviours such as the basic idea that benefits from resources need to outweigh the costs of harvesting and managing them, while effective punishment mechanisms can alter pay-offs to de-incentivize free-riding. Effective dispute resolution mechanisms can play a role in preventing individuals entering into ‘death spirals’ whereby free-riding or perceived errors are automatically responded to by withdrawing from cooperative behaviours by the other party [21]. Other elements relate to how groups decide on the rules. Collective outcomes are thought to be better when users of the resource are able to decide on the rules that affect them. This helps ensure that pay-offs are well-structured and provide incentives for individuals to continue cooperative behaviour, rather than being skewed or misaligned if others set the rules. The design principles were developed for understanding the specific context of collective management of natural resources and different features and mechanisms may be more important in other ecological or social contexts. However it seems likely that monitoring, sanctioning and dispute resolution are likely to occur in wide range of situations [22]. The design principle relating collective choice arrangement also appears to match well to arguments made in economics that institutions which enable the majority of the population to take part in economic and political decisions (‘inclusive’ institutions such as democratic voting, the rule of law) do better in terms of development, than societies that have more ‘extractive’ institutions, where a limited number of elites do well at the expense of the majority of the population [4].

Humans have created societies that are organized, controlled or coordinated on a scale not seen in other species. Common mechanisms proposed to explain cooperation in biological systems and how it has come to evolve, such as kin selection [23] or classical forms of direct reciprocity in dyadic interactions [24,25] may be important in explaining some aspects of human cooperation but they run into difficulties when trying to explain large-scale cooperation in situations involving genetically unrelated individuals, multiple individuals rather than dyadic interactions, or interactions that may not be regularly repeated [6,26,27]. Institutions may play a role in understanding how cooperation or collective behaviour is achieved [2,3] yet institutions are not an alternative to evolutionary explanations. Rather they provide the specific means by which general principles that are commonly invoked in models of the evolution of cooperation such as repeated interactions, positive assortment, or punishment [6,22,28] can be implemented. The kind of research carried out in social science disciplines described above is therefore useful in understanding how cooperative and collective behaviour can be achieved and how this varies across different societies. However, such research has often lacked a shared understanding of general processes of change that shape institutional diversity across space and time [1]. In the next section, we attempt to connect institutions to concepts from cultural evolutionary theory, and argue that this framework can play an important role in developing and testing hypotheses about institutional change.

### (c) Integrating institutions into a cultural evolutionary framework

Conceptually, we view culture as a system of inheritance that enables information to be transmitted between individuals and down generations via social learning [17]. A well-developed body of theory argues that analogies can be made between cultural change and biological evolution in terms of generation of variation, inheritance, and selection, and that there are general processes at play in the transmission and adoption of cultural traits [29]. We can think of institutions as a particular aspect of culture, and the ideas of institutions-as-rules or institutions-as-equilibria can be matched to this framework. The rules aspect connects well with the generation of variation and inheritance parts of evolutionary thinking. Institutional rules are established and modified through social processes, and knowledge about rules is transmitted by social learning and inherited within populations over intergenerational periods of time [1]. Innovations can occur in institutional rules and these rules can be consciously or unconsciously adjusted or modified. As we discuss below, there are various ways in which this might occur in human societies, often involving some form of institutional mechanism itself (i.e. there are rules about how rules are made—what can be thought of as a ‘constitutional’ process [30,31]). This connects to the fact that institutions may be interrelated, and may also interact with, or rely on, other non-institutional aspects of culture which connects to the idea of institutions-as-equilibria [32]. This means the effectiveness of a particular institutional rule can play out very differently in different social or environmental contexts, which leads to the idea that there can be coevolution between different institutions, or between institutions and other aspects of culture [1].

This coevolutionary/institutions-as-equilibria perspective can have a number of implications for understanding why societies possess the institutions that they do. Institutional arrangements may not be straightforward to implement and may build cumulatively on existing institutions and cultural traits, as has been discussed and investigated with respect to the development of other aspects of culture such as technology [33]. For example, Acemoglu & Robinson [4] argue that developing certain legal institutions which mean that certain individuals are not considered above the law can be particularly important in aiding the later development of other ‘inclusive’ institutions. As well as affecting how a particular rule may play out this coevolutionary aspect also affects whether certain institutions are adopted in the first place. For example, in the current Covid-19 pandemic countries have adopted a wide variety of different rules aimed at curbing the spread of the virus, sometimes adopting measures seen in other countries sometimes developing their own. Anecdotally, the particular rules adopted, and the success of those adopted, appear to have depended upon factors such as the existing legal and political institutions (which can affect the ability to implement or enforce things such as ‘stay at home’ orders), personal or cultural attitudes and beliefs (e.g. whether government restrictions were viewed as an affront to personal liberty), demographics and population distributions, geography and climate, and wealth (at various different levels of a society). More generally the features of groups as well the features of institutions themselves may affect how institutional rules spread between groups in a manner similar to how certain context biases in cultural evolution are thought to work [17,34], e.g. countries that are doing well economically may be more likely to be copied than countries that are performing poorly. In §2b, we present an empirical example of how these kinds of processes may be important in affecting the spread of political institutions.

The final point we make here in connecting the idea of institutions-as-equilibria to evolutionary thinking is that institutions are group-level features and can create emergent properties at the group level [35]. There can be multiple stable institutional arrangements which can represent more or less effective equilibria [3]. If there is competition between groups in a meta-population of groups then processes of cultural group selection may explain why some institutions become more common [32]. This can happen either because groups with certain institutions are better able to replace or incorporate other groups, certain institutions or groups deemed to be more attractive or effective are copied, or populations ‘vote with their feet’ and migrate preferentially to groups with certain institutions [32,36].

It is important to note that the ability of humans to create institutions is itself built upon human capacities for language, social learning, and aspects of our social cognition involving shared intentionality and theory of mind [8]. However, these factors by themselves do not necessarily promote cooperation. For example, if social learning is pay-off biased, non-cooperators may be more likely to be copied owing to the fact that their short-term pay-offs are higher [37]. Similarly, the human ability to establish rules does not necessarily mean that groups will develop efficient or effective institutions [3]. Given that there is nothing inevitable about human collective behaviour it is important to understand under how and why effective institutions emerge and spread, and to test hypotheses about the processes involved in institutional change. Here, we illustrate our approach to tackling these issues by first describing empirical comparative analyses of macro-scale institutional evolution that seek to test hypotheses about the processes that have shaped some of the major patterns we see in institutional diversity both over time and across the world today. We then go on to describe how we can go about modelling institutional change based on micro-level interactions between individuals within a population. We discuss the opportunities and challenges of both kinds of analyses and finish by considering promising areas of future research.

## 2. Macro-scale empirical analyses of institutional evolution

We can investigate how and why institutions evolve by empirically testing hypotheses using data on institutional diversity from different disciplines such as anthropology, economics and political science. These data can be productively combined with concepts and analytical approaches from evolutionary biology. The added value of an evolutionary approach here is that it captures both the specific historically contingent pathways of groups, culture and institutions, and the more general processes involved in institutional change, as well as a consideration of how these processes may be shaped by ecological factors [1,38]. Here we present examples drawn primarily from our own work examining the long-term patterns and processes involved in the evolution of large-scale societies and associated institutional complexity, and also the spread of democratic institutions in more recent history.

### (a) Long-term patterns and processes of institutional evolution and socio-political complexity

One of the biggest changes in human history has been the transition from living in relatively small groups bonded by face-to-face relationships and ties of kinship and marriage, to the massive, anonymous societies we live in today [2,39]. Whereas individual roles within early human societies were relatively undifferentiated beyond certain divisions of labour owing to age and gender, more recent societies are characterized by a diversity of different roles, and an increased number of formal and informal institutions that structure our interactions with others. These changes have not been uniform and there is great variation around the world, and over time, in the scale and complexity of human groups [7]. Systematic comparative analyses using both historical and ethnographic data can help test hypotheses about the patterns and processes involved in the evolution of institutional complexity.

Disciplines such as anthropology and archaeology have often proposed ideas about the evolution of human societies [40] but have not been able to test them convincingly owing to challenges in the kinds of data and analytical techniques available [41]. In order to more explicitly assess processes of change, Currie and co-workers [41,42] employed phylogenetic comparative methods derived from evolutionary biology to analyse ethnographic data on socio-political institutions from societies in Island Southeast Asia and the Pacific. This technique enables us to use knowledge about the ancestral, family-tree-like relationships between societies to make inferences about how features of past societies changed in order to give rise to the distribution of these features we see in the present day. These analyses showed that hierarchical decision-making institutions evolve through a series of incremental increases in levels of hierarchy, but with decreases in hierarchy also possible (more unconstrained, non-sequential models of evolution did not fit the data well). This supports the idea that in some cases socio-political institutional change can be cumulative, with institutions needing to build on previous developments.

**Figure 1.**
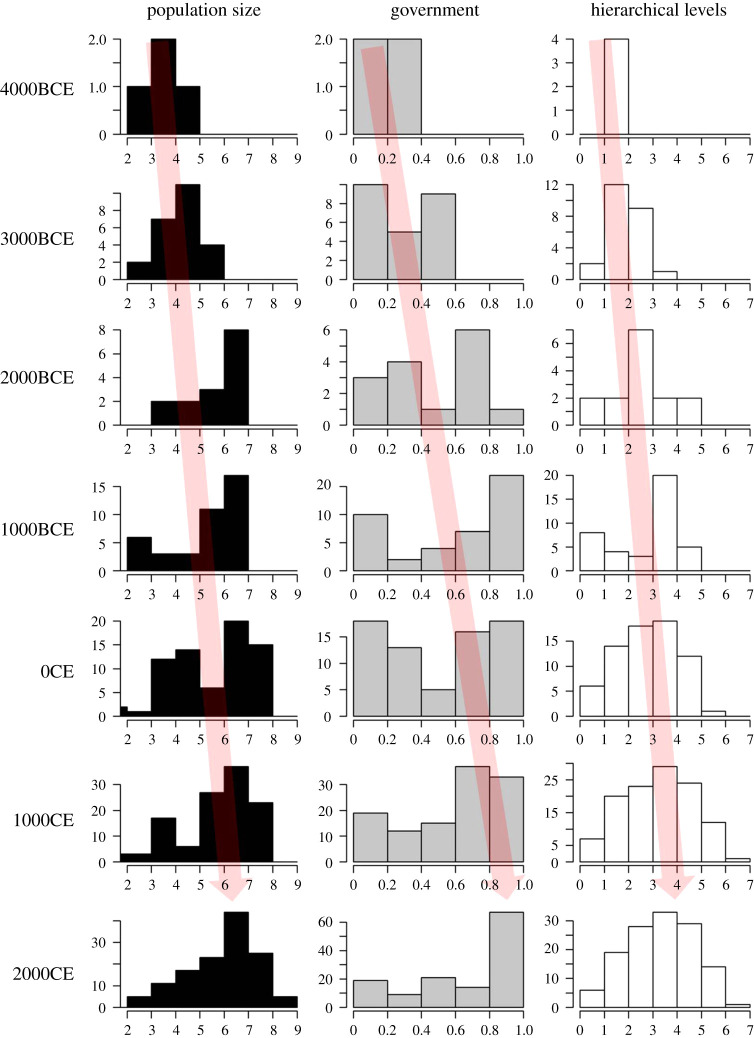
Population size, governmental roles and hierarchical levels of authority show correlated increases over time, i.e. as societies get bigger they also tend to get more complex in terms governmental and hierarchical institutions. The rows refer to the 1000-year time period prior to the dates indicated on the left. Frequency distributions are calculated based on cases drawn from a global, historical database [39]. *x*-axis values refer to log_10_ polity population size (left), governmental roles index (proportional measure, 0: no such roles, 1: maximum possible recorded in sample), mean number of hierarchical levels (see the electronic supplementary material and [39] for details of dataset and measurements). The red arrow highlights the shift in the mode of these distributions towards greater scale and complexity which is indicative of a driven macro-evolutionary trend mechanism, suggesting there is some evolutionary force favouring larger, more complex societies [43] (see text). Note also the reduction in the relative frequency of smallest, least complex societies. (Online version in colour.)

Related analyses [41] also showed that different institutional features evolve together, with increasing levels of hierarchy co-evolving with institutionalized differences in social status. Societies where decision-making extends beyond the level of a single village are more likely to have distinct and often inherited social classes (e.g. ‘nobles’ and ‘commoners’) than societies only organized at the village level. Turchin *et al*. [39] further examined these issues by constructing a global scale, time-series database going back more than 10 000 years. Using data coded from historical and archaeological information, analyses show that different aspects of societies (roles in government, number of hierarchical levels, information systems, infrastructure, economic systems) tend to evolve together, with a greater number of roles and more complex institutions emerging as societies increase in size (figure 1). The coevolution of institutions and other traits suggests that are functional relationships between different aspects of societies that enable societies to coordinate the actions of increasingly larger numbers of individuals. For example, Spencer [26] argues that the need for differentiation in political roles is spurred when a polity expands to control more territory and more people, meaning that a leader is not able to perform all the necessary roles across a large enough area by themselves. To avoid giving too much power to subordinates a new institutional arrangement needs to be created whereby leaders give subordinates control over only certain aspects of society (e.g. land managers, tax collectors, military officials).

These analyses also used information about the nature and direction of institutional change in order to reveal information about other processes involved in change. Firstly, while the overall trend has been for increasingly complex societies to emerge over time, both the phylogenetic comparative analyses and the global scale time-series analyses reveal that reductions in scale and complexity also occur. This fits with the idea that establishing appropriate institutions to meet a new adaptive problem is challenging, and even with the human ability to design and plan we cannot always find appropriate solutions [3]. The historical and archaeological records show many instances of societies decreasing in size because they did not develop new institutions to control larger areas, or adapt to changes in ecological or social conditions [27–30]. Societies may go through several cycles of expansion and collapse [31], potentially with new rules and systems of organizing relationships being experimented with (sometimes building cumulatively on previous attempts) until effective solutions are found [44].

While decreases in complexity do occur, in these analyses increases in complexity appear to be more common than decreases. This appears to be consistent with what macro-evolutionary theorists in biology would label a driven evolutionary trend, in which there is a directed, pervasive force that favours movement of trait values in a particular direction [43]. This is as opposed to a passive trend, where there is no overall bias towards changes in a particular direction but instead an overall direction is apparent because species or societies emerge initially at or near the lowest possible value. Evidence for a driven trend is also apparent when examining changes in the frequency distribution of societies in terms of their size and institutions (e.g. number of governance roles and levels of decision-making; figure 1) [39]. This shows not only a change in the right tail of these distributions (which would be seen in both passive and driven trends), but also a shift in the peak of the distribution towards increasing complexity over time i.e. not only do larger, more complex societies become more frequent over time, but smaller, less complex societies become noticeably less common.

If there is a driven trend, then we can ask what the evolutionary process that appears to favour increasing social scale and complexity might be. One possibility is that individuals within groups are more motivated to expand groups and potentially gain more resources than they are to stabilize or down-size. Another potential explanation is that competition between groups, particularly in the form of warfare leads to a form of cultural group selection (CGS), with the link between warfare and institutional complexity potentially occurring via two processes. Firstly, when a group conquers another group and attempts to incorporate it, this potentially leads to the kinds of challenges in organizing individuals discussed above which then favours the development of novel institutions to make the expanded group more stable. Secondly, groups that have institutions which enable them to be larger or better coordinated are better able to defeat smaller or less efficiently organized groups. In order to test such an idea, Turchin *et al*. [45] developed a spatially explicit agent-based model in which selection favoured larger groups and therefore also favoured the evolution of institutions that enable groups to maintain larger size. Simulations were run on a landscape reflecting the Afro-Eurasian landmasses with the intensity of warfare being influenced by the historically attested spread of horse-based military technologies from the Eurasian steppe. Outputs from the model showed a good match to data on the historical distribution of large-scale societies from 1500BCE to 1500CE, suggesting this mechanism is at least a plausible explanation.

More recent statistical analyses of the historical distributions of large-scale societies [46] show that in addition to support for the predictions of the steppe-warfare hypothesis, there is also support for the idea that regions in which agriculture has been practised for longer have tended to be home to larger scale societies for longer [47,48]. This again is consistent with the idea that the development of effective socio-political institutions is a cumulative process, i.e. these regions have had more time to find solutions that work and develop the kinds of institutions that enable societies to function on a larger scale. Other comparative analyses also suggest that these long-term historical and ecological factors may explain some of the variation we see in the effectiveness of societies in the modern world. Using cross-national data and path analysis, Flitton & Currie [49] tested different potential pathways by which ecological factors could have affected historical development of socio-political institutions, which in turn may have shaped modern institutions and economies. Analyses show that countries with more ‘inclusive’ modern-day institutions tend to have higher gross domestic product (in line with ideas discussed in §1 [4]). However, this measure of institutional quality is itself predicted by a measure of how long the region where a country is located has had complex governance beyond the local level. This measure of ‘State history’ is in turn predicted by timing of agriculture (how long has agriculture been practised in the region), which is itself predicted by an ecological variable (latitude). These analyses show the value in using cultural evolutionary theory as a framework to bring together different ideas about social evolution and explicitly assess which hypotheses receive support and which do not.

### (b) Coevolution and the spread of institutions

The understanding that institutions evolve within societies, and that they co-evolve with other institutions and other aspects of culture has implications for how institutions might spread between societies. As discussed above, the effectiveness of institutional rules may be supported by other institutions, values or norms such that simply taking a rule from one society and transplanting it to another means the rule may not work as well or may lead to unintended consequences. A prediction stemming from this idea is that institutions may spread more easily between societies the more closely related they are culturally. Some economists, inspired by evolutionary theory, have employed such logic to assess the spread of innovations that help create economic growth [50]. This idea is similar to the ways that pathogens can spread more easily between genetically similar hosts [51], and here we show how we can adapt phylogenetic methods from evolutionary biology to test such ideas.

**Figure 2.**
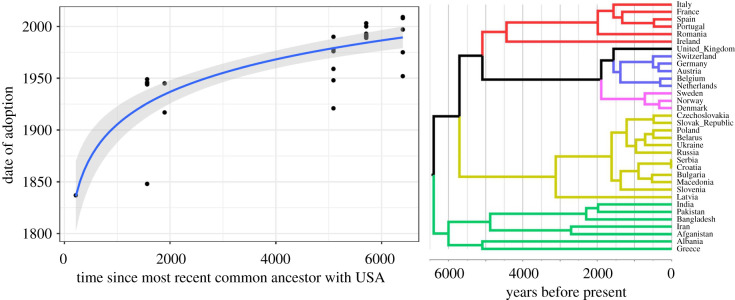
Countries that are more culturally distant from the earliest modern democratic country (USA) have tended to adopt open and competitive election institutions later than culturally similar countries (data taken from [52]). Cultural distance is based on time in years since the most recent common ancestor (MRCA) measured on a phylogenetic tree of Indo-European languages [54] (right). Where countries have adopted and abandoned elections we use the last calendar year that democratic election institutions were adopted (*y*-axis). Coloured (non-black) branches on the phylogenetic tree represent groups that have the same MRCA with the USA, which relate to the points on the left that share the same values on the *x*-axis. (Online version in colour.)

To illustrate the approach, we can examine the spread of modern democratic voting institutions since the year 1800 using the POLITY V dataset [52] and match this with a phylogenetic tree that acts as a proxy for deep historical and cultural relatedness between societies, i.e. societies that speak more closely related languages will tend to be more similar in other cultural and institutional features [53]. This technique relies on suitable phylogenetic information being available, so for these preliminary analyses we confine our dataset to countries in Eurasia speaking Indo-European (IE) languages in order to make use of a phylogenetic tree of IE [54] that has been used in other cultural evolutionary studies [41]. The earliest democracy in this dataset is the USA and is considered the source of the spread of modern democratic institutions to other countries. Figure 2 shows that countries that have a shorter cultural distance from the USA (time in years since the most recent common ancestor measured on the IE phylogenetic tree) tended to adopt and retain open and competitive elections earlier than more distantly related countries. There are several cases of countries losing then re-adopting democratic institutions and therefore these data only consider the last instance that democracy was adopted. However, a qualitatively similar pattern is also found if we use the earliest dates that democratic elections were adopted (see the electronic supplementary material). This is consistent with the idea that societies more closely related historically are likely to have other cultural traits that make the adoption of new democratic institutions more stable. Interestingly, there appears to be a stronger relationship when considering the last date of adoption rather than the first date of adoption. This suggests that cultural distance is affecting how well new institutions play out rather than simply being an indicator of how likely the institution is to be adopted in the first place (although this may also be important). This finding is consistent with analyses by Spolaore & Wacziarg [55] who show similar effects of population distances and differences in democratic institutions as well as other measures of ‘inclusive’ institutions (Repudiation of Contracts, Risk of Expropriation and Rule of Law) using a global sample and controlling for other potential geographical factors. Our current findings are still somewhat preliminary and we will be expanding the countries and variables covered in an attempt to assess different hypotheses in future. However, these analyses help illustrate the logic of this approach and the potential added value of incorporating an understanding of cultural evolutionary history and processes into analyses of institutional diversity.

## 3. Developing micro-level models of institutional evolution

The examples above illustrate the value in testing hypotheses about institutional evolution using macro-scale data. The ideas tested in such analyses relate to both processes occurring within populations (micro-level) and also processes occurring above the population-level (macro-level), e.g. between group competition, borrowing. In many ways, the macro-level processes are similar conceptually to existing models of cultural evolution. However, different factors or processes may be important at different scales [56], and micro-level processes may have consequences for how macro-scale processes play out. For example, if macro-evolutionary processes like CGS depend on emergent properties of individual interactions [35], then how these emergent properties are realized may have consequences for whether selection at the group level is working against individual selection or whether it is amplifying or enhancing it [32,57,58], which can have consequences for the rate at which traits may change in frequency in the meta-population of groups. To date there has been relatively little development of formal models of institutional evolution at the micro-level that is based on behaviour of individuals and the interactions between them [2]. Perhaps because they appear to be peculiar to humans, institutions have received relatively little attention in evolutionary models of cooperation. On the other hand, most current models of cultural evolution focus on transmission of information between individuals. These approaches have provided many important insights into understanding human behaviour; however, this approach does not capture the social processes by which rules are generated or agreed upon in the first place, and the evolutionary consequences of different processes at both the micro and the macro level.

To illustrate one way we might go about developing individual level models of institutional change we present a simple agent-based model. We follow the suggestion of Powers *et al*. [2] by adopting a two-stage game-theoretical approach. This approach is itself based on models developed in economics ([31], see also [59,60]), but as of now has only been applied relatively rarely in models of human social evolution (e.g. [61]). In normal game theory models, agents play an ‘economic game’ with certain pay-offs for different behaviours. To model the role that institutions play, we have our agents play a ‘political game’ that sets a rule that agents follow that has consequences for the economic game. Here we use the familiar situation of punishment for non-contribution in a public goods (PG) game [62]. The main feature of this model is that the system starts with no punishment and agents have the ability to introduce this by being able to set and agree on a punishment value that can be updated and adjusted through agents' actions throughout the simulation. To keep things simple, we have attempted to minimize the presence of other institutional features such as a centralized mechanism for punishment, status differences or official positions of leadership (see §3c for further discussion on this and other related points). We stress that the model presented here is primarily for illustrative purposes and below we discuss some of the reasons for the particular modelling choices, some of the limits or challenges of such an approach, and also the ways in which it might be modified or updated.

### (a) Model set-up

Details of the model are given in the electronic supplementary material but we summarize the key conceptual points here. In this model, the economic game involves agents contributing a set amount (10%) of their budget (10 units) to a public pot. Any donations are then doubled and redistributed to all agents. This presents the classic collective action problem [63]: the biggest overall benefit comes if all agents contribute their full amount, yet individually an agent will get a bigger pay-off by not contributing and yet still receiving a share of the public good. If agents are able to choose their strategy based on achieving the best individual pay-offs, or if the game is played over multiple generations and strategies are heritable, then those that cooperate by donating to the public good will reduce in number compared to those who free-ride on the contributions of others.

In our model, we consider a population of agents with fixed strategies in the economic game (i.e. they cannot change their behaviour), who will either donate to the public good (cooperators) or not (free-riders). Our agents play the economic game and receive their portion of the public good. In our political game, agents have an ability to set a value for a fine for being a free-rider. Our simulation starts with this value being zero, which is equivalent to no punishment. If fines are introduced by the population (i.e. punishment is decided to be set greater than 0), free-riders are punished by selecting an equal number of other agents from the population to act as obligate punishers (both cooperators and free-riders in the economic game can be chosen as punishers). Those selected as punishers for that round pay the cost of punishing and free-riders pay a cost that is twice this amount. In the political game, at each generation one new value for the level of punishment is proposed, with a number drawn randomly from a normal distribution with a given standard deviation. The group of agents then ‘vote’ to decide on whether they will accept the new level of punishment or stick with the previous level. Their decision is based on assessments about the long-term difference in pay-off they will receive as individuals under both scenarios. After playing the PG game and performing any punishment pay-offs are calculated. These pay-offs then affect individuals' probability of reproducing and forming part of the next generation (group size is fixed). Increasing punishment values above a certain level decreases net pay-offs to free-riding and will reduce the proportion of free-riders in the population over time. Here we want to show what happens when we start with a zero level of punishment and a very low proportion of cooperators in the population (5%) to see if punishment can be introduced, leading to cooperators increasing in frequency over time.

### (b) Model results

**Figure 3.**
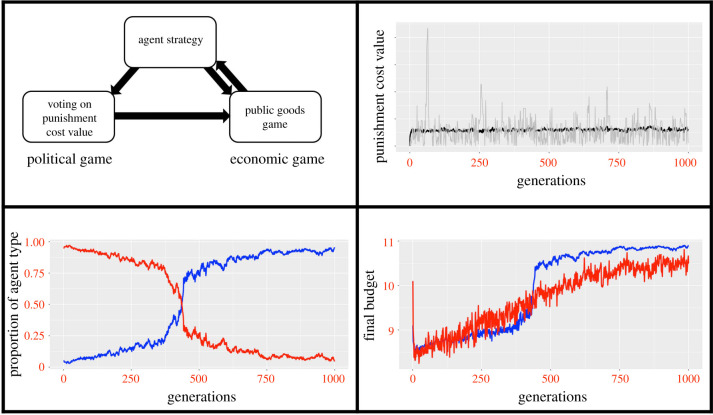
Output from an agent-based simulation implementing an institutional form of punishment in a public goods game. A schematic (top left) shows how the model works. Agents have a fixed strategy of either being cooperators or free-riders in the economic game, and vote to decide on a level of punishment for free-riders (the political game). Pay-offs from the economic game determine agent fitness and thus feedback to affect the proportion of different strategies in the next time step of the simulation. The median cost value (black line) over 1000 generations of 100 simulation iterations is shown (top right), with an example from one iteration (grey line) to illustrate the kinds of dynamics seen in the model. The punishment cost value affects the pay-offs to agents in the economic game, giving cooperators (blue) higher pay-offs than free-riders (red) (bottom right), leading to changes in the proportion of cooperators such that they become the most common strategy (bottom left). Agents vote for punishment values that although they lead to lower pay-offs in the short term, in the longer term lead to pay-offs that are better for cooperators and any free-riders in the population (bottom right). Values shown in bottom graphs are medians from 100 simulation iterations.

Figure 3 shows the output from 100 iterations of the model and shows how the mechanism works. Punishment values start at 0, until such point that a value is proposed that leads to (i) an immediate higher pay-off to cooperators, and (ii) a better predicted long-term pay-off for free-riders (who are in the majority at that point) compared to the prediction based on the current cost. The increased relative pay-off to cooperators means that free-riders are removed from the population over time. Over time the pay-offs to cooperators and free-riders increase (the remaining free-riders benefit from the fact that there are more cooperators around so the PG is greater). Once cooperators have been established as the majority in the population occasionally punishment values are accepted that lead to free-riders increasing in frequency until a new value is proposed that restores the relative advantage to cooperators. This particular mechanism (with its associated assumptions) is therefore able to promote and stabilize cooperation under these conditions. In the electronic supplementary material, we present the results of some sensitivity analyses which show that as long as agents are able to propose punishment values that are sufficiently high then free-riders will be removed from the population.

### (c) Model discussion: limitations and future extensions

This model is not intended to be a complete or even a realistic description of how real-world institutions work but it does serve to illustrate some important points. The model demonstrates the logic of the two-step political-game-then-economic-game process, and the ways that institutions can be socially determined and go about altering pay-offs. In our example, agents were able to overcome the ‘default’ set-up of the PG game, and its associated collective action problem, by adopting values of punishment that are sufficient to reduce the frequency of free-riders but are not so high that they outweigh the benefit this brings. Constructing the model helps bring focus to various issues around the nature of the political game and how agents use information and make decisions. Here we discuss some of the limitations of the current model and outline ways that the approach will be developed in the future.

Firstly, in this model individuals that are selected to punish have to carry out punishment (i.e. we do not allow individuals to decide whether they can punish, or have fixed strategies of ‘punisher’ or ‘non-punisher’). This means that we are in-effect side-stepping the second-order free-rider problem—because punishment is costly individuals would receive a higher pay-off if they did not punish. Such automatic, reflexive behaviour can potentially come about through some kind of internalized norm, but that would simply beg the question of how such a norm came to be. More realistic models of punishment will be developed in the future that allow for the possibility of non-punishment. Similar to mechanisms such as ‘strong reciprocity’ [64] some form of group selection where in groups with higher frequencies of non-punishers would be more likely to go extinct is one possible solution to the problem [22]. However, as Powers *et al*. [2] point out this is not the only solution and in fact examples from the real world (such as the examples described in §1) show that certain institutional rules can lead to direct benefits for punishers (see also [58]). In contrast to the peer-punishment system we have used here other models of institutionalized punishment have employed a pool-punishment system whereby a proportion of an agent's returns from the public good goes into a central pot that is used to finance punishment [61,65]. We chose to start with a peer-punishment mechanism as this arguably requires fewer additional elements in comparison to a population that lacks any form of institutionalized punishment (see also [66] which has a peer-punishment mechanism but incorporates individual heterogeneity). An additional consideration is that a centralized punishment mechanism has to be financed which may be more or less difficult under different conditions. A centralized, pool-punishment system is often associated with formal offices of leadership, which is not a common feature of the kind of egalitarian, forager groups that characterized much of human history.

This discussion also raises issues about the decision-making process and set-up of the political game aspect of our model. Here we chose a majority-rule voting mechanism in order to have acceptance of a new punishment value, which we envisaged abstractly as capturing discussions that groups might have in order to gather individual viewpoints as seen in many small-scale group situations, rather than necessarily a formal vote that we see in modern, democratic settings. Although we feel it would be unlikely to dramatically affect the results of the current model, more explicitly deliberative decision-making processes may be relevant for many informal institutions, e.g. Perret *et al*. [67] and Gavrilets *et al*. [68] explicitly model consensus decision-making processes. It is also worth pointing out that having a recognized system for agreeing upon rules is an institutional feature itself and so our model assumes this mechanism is in place. Furthermore, in our model institutional change occurs via deviations in a single, continuous parameter. However, institutional changes may occur in other ways that could be modelled in future work such as introducing a new type of rule, changing who a rule applies to or other conditions about the rule, or adding additional rules. We return to this point in the conclusion.

Other key assumptions in our model are that all individuals have the same ability to influence the outcome of the political game, and that the agents playing the political game are the same as those playing the economic game. Both conditions are probably present in small-scale, egalitarian groups such as those that characterized much of human history. Furthermore, as we discussed in §1 the ability of resource users to set their own rules is a feature of groups that are able to effectively manage commons. However, as discussed above some of the most striking patterns of institutional evolution we see in human history involve changes in hierarchical decision-making processes and the degree to which different individuals within a society are able to influence institutional rules. An area that will be explored in future will be to build on some existing models of the evolution of inequality and hierarchy [69–73] to model situations where different agents have different levels of influence or power in deciding institutional rules. These differences in socio-political systems may have a number of important effects on the nature and dynamics of social evolution including the stability of groups, or the effectiveness of collective action either in different realms such as resource management or between-group competition.

Finally, we can consider the nature of the agents themselves and the potential effects this has on institutional evolution. Our agents can only play fixed strategies in the economic game and thus the proportion of agents playing ‘cooperate’ can only change via selection when agents reproduce. Given the fixed-strategy nature of our agents, in order to make decisions about how to vote in the political game that would allow them to receive higher future pay-offs they were imbued with an ability to make accurate long-term predictions about the consequences of different punishment values. While fine for illustrating the logic of the two-stage modelling process, these assumptions are not very realistic for humans. In reality, people have the ability to modify behaviour according to circumstances, but do not have perfect information or unbounded cognitive abilities. Some degree of foresight in assessing the likely consequences of different outcomes seems a reasonable assumption for many situations in which groups may decide on institutional rules. Recent models [65,66,70] have shown how limited foresight mechanisms which individuals use to account for future pay-offs and how others are likely to respond to one's own strategy can help groups to solve collective action problems. The knowledge, abilities, biases and other features of agents are things that can be incorporated into future models, and a valuable aspect about taking an evolutionary approach is that these properties do not have to be treated as fixed but can themselves be modelled as evolving either genetically or culturally depending on the context being addressed.

## 4. Conclusion

Incorporating institutions more explicitly into cultural evolutionary theory is an important challenge for establishing a more complete understanding of human collective behaviour and the diversity of human societies. Here, we have discussed the logic behind the evolutionary function of institutions, the processes involved in shaping some of the broad patterns we see in institutional evolution, and illustrated how we might model the evolution of institutions based on interactions between individuals. Many key issues relating to understanding institutional evolution remain for future research. We have argued that the social processes involved in institutions are somewhat different to most existing models of cultural evolution. However, we have also stressed that knowledge about rules is transmitted by social learning processes as are other norms, beliefs and values that can affect how institutions work. Understanding how these different processes interact is an important area of research especially as some existing models suggest that pay-off biased social learning may work against cooperative behaviour [37]. Furthermore, we have not focused here on the proximate mechanisms that affect how individuals decide on or interact with rules, or how they learn these rules during their lifetime but these are worthy of future consideration and are important to address within an integrated evolutionary framework [1,3,74].

Institutions are group-level feature of human societies, and here we have attempted to outline how we can approach the study of institutions both by examining between-group features in institutions and understanding the social interactions between individuals that give rise to institutions and their group-level effects. The simple model we introduced in §3 deals with gradual changes in a narrowly defined aspect of a particular type of institution. However, the ability to consciously design or plan new institutions or amend rules in a targeted way means that larger, or more fundamental changes in institutions and social systems can be introduced that do not rely directly on the kinds of micro-level evolutionary processes that cultural evolutionary theory has traditionally concerned itself with. For example, in the late nineteenth century Japan dramatically changed its institutional arrangements dramatically from those of a feudal system with the Shogun at its head, to those that seemed more aligned with a European industrialized political and economic system [75]. In future research, it will be important to model other kinds of processes of institutional change.

The ability to consciously plan or design institutions does not necessarily mean they will be effective, persist and lead to stable social systems. As discussed above groups may try different institutional arrangements before they find something that works. Approaches to modelling more discontinuous institutional change will also benefit from a broader understanding of the context in which such changes take place, as this may affect the success of new institutions. For example, even when change does appear to be discontinuous it may be owing to the adoption of pre-existing institutions from other societies and thus not be completely novel. The success of adopting particular institutions may involve repurposing or adapting existing institutions. For example, in the case of Japan, Western societies acted as a template for the system but they also used and built upon traditional Japanese institutions such as installing the Emperor as a head of state in a manner similar to many European countries [75]. Our example above of the spread of democratic institutions illustrates the potential application of cultural evolutionary theory and methods to understand some of the general processes that may influence the adoption of new institutions from other groups and this approach can be extended in many ways to address different hypotheses not just those based on cultural similarity. As the ways in which institutions change is large, one approach to modelling may be to develop models that are inspired by certain, specific types of institution. For example, Aktipis and colleagues have developed models based on the institution of risk-pooling in East African Maasai communities known as Osotua, that not only provides insights into this specific feature but also cooperation and risk-pooling in variable environments in general [76].

This discussion also draws attention to an important distinction between institutional change in terms of the processes by which change occurs within a society, and the consequences of these changes in any meta-population of groups that might lead to certain institutions increasing in frequency over time. An area of debate that is of particular relevance to institutional evolution is the importance of CGS as a mechanism for achieving collective action [3,77]. Theoretical work has demonstrated the logic of the idea, and empirical work has shown that many of the assumptions of such models are met [32] (but cf. [78]) making it a plausible explanation. However, conversely there is recognition that term CGS has been used in slightly different contexts [79], which is important as different mechanisms can result in different evolutionary dynamics [36]. Furthermore, as discussed above, institutions can lead to solutions to cooperation or coordination problems without an explicit mechanism involving differential group death, copying or migration [2]. We will be developing agent-based models as a means of explicitly addressing some of these debates, and as we describe in §2 it is also important to formally test the predictions of CGS hypotheses against potential alternative explanations in order to assess how well they might explain real-world examples of institutional evolution [80].

This focus on processes of change and the ability to link micro-level processes and macro-level processes and outcomes is where an evolutionary perspective can potentially be most informative to approaches developed in other disciplines. For example, the kinds of approaches taken in sustainability science described in §1 have had many important insights into the functional design features that make for effective institutions, yet have paid less attention to how these features emerge in the first place, or what affects their dynamics over time [81,82]. Currently, we are working with communities in northern Kenya to understand how the institutions for collective action in a pastoralist system are playing out across a landscape of different cultures and environments [83]. Given the highly variable nature of rainfall in this environment, individual private ownership of small areas of land is not effective, and traditionally pastoralist communities had collective systems of land tenure [84]. Over the past couple of decades, in response to increased pressure on the rangelands, communities in this region have been establishing new organizations (conservancies) designed to manage their land in a more sustainable manner [85]. The aim of the governance institutions of these new conservancies is to improve the collective-choice arrangements by making them more inclusive and improve the representation of women and young men. We are working with the communities and associated organizations to understand how cooperation is achieved in these communities, how effective the new institutional rules are at achieving collective action, and what social and environmental factors affect the success of conservancies. One prediction, which is related to the ideas about the coevolution and spread of institutions discussed above, is that new institutions may be more likely to be adopted and work effectively when they more closely match existing social norms and institutions. Understanding such processes may help in efforts to more efficiently spread effective approaches to natural resource management by suggesting ways they can better fit with social and ecological contexts. Increasing our understanding of how and why institutions evolve is therefore not only of theoretical interest but has important implications for a range of applied issues too.

## Data accessibility

NetLogo and R code relating to simulation model are available from the University of Exeter Repository—Open Research Exeter http://hdl.handle.net/10871/125446. The modified Indo-European Language tree discussed in §2b is included in the electronic supplementary material. Other data are described in the electronic supplementary material and are freely available from existing sources referenced therein.
